# Acetylation of MnSOD directs enzymatic activity responding to cellular nutrient status or oxidative stress

**DOI:** 10.18632/aging.100291

**Published:** 2011-02-28

**Authors:** Ozkan Ozden, Seong-Hoon Park, Hyun-Seok Kim, Haiyan Jiang, Mitchell C. Coleman, Douglas R. Spitz, David Gius

**Affiliations:** ^1^ Departments of Cancer Biology, Pediatrics, and Radiation Oncology, Vanderbilt University Medical Center, Nashville, TN 37232, USA; ^2^ Free Radical and Radiation Biology Program, Department of Radiation Oncology, Holden Comprehensive Cancer Center, The University of Iowa, Iowa City, IA 52242, USA

**Keywords:** MnSOD, Sirt3, mitochondria, acetylation

## Abstract

A fundamental observation in biology is that mitochondrial function, as measured by increased reactive oxygen species (ROS), changes significantly with age, suggesting a potential mechanistic link between the cellular processes governing longevity and mitochondrial metabolism homeostasis. In addition, it is well established that altered ROS levels are observed in multiple age-related illnesses including carcinogenesis, neurodegenerative, fatty liver, insulin resistance, and cardiac disease, to name just a few. Manganese superoxide dismutase (MnSOD) is the primary mitochondrial ROS scavenging enzyme that converts superoxide to hydrogen peroxide, which is subsequently converted to water by catalase and other peroxidases. It has recently been shown that MnSOD enzymatic activity is regulated by the reversible acetylation of specific, evolutionarily conserved lysine(s) in the protein. These results, suggest for the first time, that the mitochondria contain bidirectional post-translational signaling networks, similar to that observed in the cytoplasm and nucleus, and that changes in lysine acetylation alter MnSOD enzymatic activity. In addition, these new results demonstrate that the mitochondrial anti-aging or fidelity / sensing protein, SIRT3, responds to changes in mitochondrial nutrient and/or redox status to alter the enzymatic activity of specific downstream targets, including MnSOD that adjusts and/or maintains ROS levels as well as metabolic homeostatic poise.

## INTRODUCTION

One theme that has emerged in the last several years is that aging, perhaps better defined as longevity, is a complex genetic and cellular process that appears to be regulated, at least in part, by a relatively new gene family referred to as sirtuins [1,2]. These genes are the human and murine homologs of the *Saccharomyces cerevisiae* Sir2 that has been shown to regulate both replicative and overall lifespan [3,4]. The sirtuin family genes have also been shown to direct longevity in *C. elegans* and *D. melanogaster* [1,2] suggesting an evolutionary conserved need for these proteins in the cells in multiple different complex species. Sirtuins are classified as class III histone deacetylases, which are different than traditional class I and II histone deacetylases [5,6]. Unlike conventional HDACs, sirtuins have a variety of substrates ranging from metabolic enzymes to structural proteins as well as histones [7,8].

The mammalian sirtuin family consists of seven NAD^+^-dependent protein deacetylases [9-11] that share a common 275-amino acid catalytic domain and are localized to the nucleus (SIRT1, 6, and 7), mitochondria (SIRT3, 4, and 5), and cytoplasm (SIRT2), respectively [3]. Sirtuins require NAD^+^ as a cofactor which mechanistically connects their enzymatic activity to the metabolism of the cells, and provides link between the sirtuin activity and energy and stress responses [12,13]. Using these findings as a guide, it seems reasonable to suggest that sirtuins function as fidelity or watchdog proteins signaling proteins that alter the activity of downstream targets via post-translational modifications involving protein acetylation to maintain cellular metabolic homeostasis [14].

In this regard, lysine acetylation has recently emerged as an important, and perhaps the primary, post-translational modification employed to regulate mitochondrial proteins [14,15]. Several proteomic surveys have identified a disproportionately high number of acetylated proteins in the mitochondria and many of these proteins are associated with energy homeostasis [14,16]. Thus, these results suggest that the mitochondrial acetylome, which is regulated by both acetyl transferases as well as deacetylase such as sirtuins, may by the primary means by which the mitochondrial regulates energy production and ROS levels to match ATP production with intracellular energy requirements. In this regard, three of the seven mammalian sirtuins appear to be localized to the mitochondria [11], including SIRT3 that is the primary mitochondrial deacetylase [17], suggesting a significant role for these sirtuins in regulating mitochondrial metabolism [12,17]. Thus, we and others hypothesized that SIRT3 may act as a maintenance proteins that significantly determines the mitochondrial acetylome that functions by monitoring critical mitochondrial processes such as the regulation of respiration and/or metabolite clearance, and initiating a protective and/or reparative response.

Based on these results, it seems reasonable to propose that acetylation of mitochondrial proteins may play a role in maintaining and regulating mitochondrial ROS levels as well as function. Sirt3 is the main mitochondrial deacetylase [17], and genetic knockout of Sirt3 results in increased ROS, including superoxide levels, *in vitro* and *in vivo* [12]. Thus, we believe it is a logical extension to hypothesize that SIRT3 is a regulatory protein, maintaining mitochondrial homeostasis via changes in the acetylation of metabolic target proteins, including those comprising the mitochondrial ROS detoxification system.

We have previously demonstrated that MEFs lacking *Sirt3* exhibit an immortalization permissive phenotype and the knockout mice spontaneously form well differentiated, estrogen and progesterone (ER/PR)-positive mammary tumors [18]. SIRT3 protein levels are decreased in human breast cancers as well as several other human malignancies [18] suggesting that SIRT3 is a genomically expressed, mitochondrial localized tumor suppressor. In addition, it has also been shown that the Sirt3 knockout mice are permissive for other age-related illnesses including fatty liver [13], insulin resistance [19], and cardiac hypertrophy [20,21]. These results identified Sirt3 as a more generalized mitochondrially fidelity protein and the mice lacking Sirt3 may be useful *in vivo* models to investigate human illnesses. One intriguing finding from all of these manuscripts is that cells lacking Sirt3 exhibited altered mitochondrial metabolism as exhibited by increased mitochondrial ROS and superoxide levels during stress. These results suggest that the aberrant regulation of the mitochondrial acetylome, which by definition occurs in murine cells lacking Sirt3, results in a phenotype permissive for mouse conditions that mimic human illness as well as suggest an underlying mechanism involving increased ROS and superoxide levels.

### MnSOD as a Sirt3 deacetylation target that directs enzymatic detoxification activity

Manganese superoxide dismutase (MnSOD) is one of the primary mitochondrial antioxidant in a network of detoxification enzymes that neutralizes the highly reactive superoxide ions (O_2_•^-^) to less reactive hydrogen peroxide (H_2_O_2_) followed by its immediate conversion to H_2_O by catalase and other peroxidases in the mitochondrial matrix [22]. Superoxide is the by-product of electron transport chains I and III while they work inefficiently, and arises as a primary damaging reagent for mtDNA and other mitochondrial macromolecules [19,23]. ROS, in order of sequential reduction from O_2_, include superoxide (O_2_•^-^), hydrogen peroxide (H_2_O_2_), hydroxyl radical (.OH), and organic peroxides, that are normally produced during respiration [24]. While low amounts of ROS are natural side products of various electron transfer reactions and easily tolerated by the cell, abnormally high levels of ROS from any number of possible sources induces oxidative stress and can damage cells by peroxidizing lipids, and disrupting proteins and nucleic acids [25,26]. Since MnSOD enzymatically scavenges superoxide, which is increased in cells lacking Sirt3 [27], it seemed logical to suggest that cells lacking Sirt3 might have altered regulation of MnSOD activity.

The balance between the mitochondrial antioxidant detoxification system and ROS should be finely maintained while low levels mitochondrial ROS in are required by cells to modulate normal redox signaling networks. In addition, excessive amounts of ROS is believed to shortened life span [28] and induced age-associated pathological conditions, such as carcinogenesis, cardiovascular, and other diseases [29,30]. While MnSOD^-/-^ mice are neonatal lethal the MnSOD^+/-^ mice display higher oxidative damage and incidence of tumor formation [31,32]. Mutations in SOD2 were associated with human age-related disorders, such as cardiomyopathy, neuronal diseases, and cancer [33-36]. These results clearly demonstrate the significant role for the aberrant regulation of MnSOD in age-related illnesses and suggest that upstream signaling proteins that regulate MnSOD may also play a role in these pathologies as well.

There is an accumulating evidence that in specific solid tumors there is a very strong correlative statistically connection between aging as well as aberrant mitochondrial ROS regulation [37-39]. In this regard, the mice lacking Sirt3 appear to be an new *in vivo* model to investigate the connection between aging and carcinogenesis since these mice, at ages greater than 13 months, developed well differentiated ER/PR receptor positive mammary tumors, which is the subtype of breast cancer that is most commonly observed in postmenopausal women [18]. Kim and his colleagues proposed that increased mitochondrial superoxide level was one of the major reasons triggering breast tumor development in the older *Sirt3* knockout mice. In addition to breast tissue, SirT3 deficient mouse hepatocytes and cardiomyocytes displayed significant mitochondrial superoxide levels [18,20]. Thus, it has been proposed that SIRT3 protects against ROS by enhancing the activity of antioxidant defense system, suggesting that this protein could be an imperative mitochondrial fidelity protein in the face of oxidative stress [18,20,40]. Based on these results our research group hypothesized that Sirt3 may bind to and regulate the enzymatic properties of MnSOD that detoxifies mitochondrial superoxide so as to prevent oxidative stress and cellular damage [41].

In this regard, we have recently shown in a manuscript published in *Molecular Cell* that MnSOD contains a reversibly acetylated lysine residue that is deacetylated by caloric restriction and 36 hours of fasting and MnSOD [41]. Purified MnSOD was also shown to be directly deacetylated by recombinant SIRT3 in an *in vitro* deacetylation assays and re-introduction of the wild-type, but not a deacetylation null, *Sirt3* gene decreased MnSOD acetylation as well as increased MnSOD activity. These results strongly suggested that Sirt3 deacetylation activity directly directs MnSOD acetylation status as well as its enzymatic detoxification activity [41].

It was also shown that MnSOD physically interacts with Sirt3 and lysine 122 is deacetylated by Sirt3, suggesting that acetylation of this lysine, at least in part, may regulate MnSOD function. This idea was validated by a MnSOD mutant that demonstrated increased activity when lysine 122 was changed to arginine (to mimic the deacetylated state). MnSOD^K122-R^ decreased superoxide levels in MEFs lacking MnSOD as well as prevented IR-induced transformation of MnSOD^-/-^ MEFs. Infection of MnSOD^K122-R^ into MEFs lacking*Sirt3* also decreased cellular superoxide levels and reversed the tissue culture immortalization / transformation permissive phenotype observed in these primary cells. These results suggest that MnSOD amino acid K122, which is conserved in multiple complex and primitive species, including *C. elegans*, directs MnSOD enzymatic detoxification activity as well as plays a role, at least in part, the damage permissive phenotype observed in murine cells lacking*Sirt3* [41]. Similar results for the role of Sirt3 regulation of MnSOD via acetylation has also be shown by the Chen laboratory [42] however, this group identified lysines 53 and 89 as acetylation targets that direct enzymatic function. Thus, this implies that MnSOD may contain additional reversible acetyl lysines that determine enzymatic activity.

In this manuscript we also showed livers from the *Sirt3* knockout mice exposed to 2 Gy of radiation on two consecutive days demonstrated an ionizing radiation-induced damage permissive phenotype. This was determined by radiation-induced liver periportal to midzonal hepatocellular swelling, dilation of the cytoplasm, poorly defined vacuoles, and increased apoptosis and ROS, specifically ONOO-, a reaction product of nitric oxide and superoxide [41]. This histology displays some characteristics similar to, at least in part, to that observed in microvesicular steatosis that is associated with mitochondrial dysfunction [43]. Interestingly, the risk factors for steatosis include diabetes mellitus, protein malnutrition, and obesity [43], all of which have been associated with abnormalities of Sirtuin function [11]. These results suggest an oxidative stress permissive phenotype, including increase cellular superoxide levels, in liver cells lacking Sirt3. These results as well as those observed by others for fatty liver [13], insulin resistance [19], and cardiac hypertrophy [20,21] clearly show that the mice lacking Sirt3 exhibit a cell damage permissive phenotype for age-related illnesses and strongly suggest a mechanisms involving aberrant accumulation of mitochondrial superoxide levels. Finally, while the data does not definitively show that the aberrant acetylation a subsequent regulation of MnSOD is causative in these pathological processes, these results do make a strong potential scientific argument for altered regulation of MnSOD.

**Figure 1.**
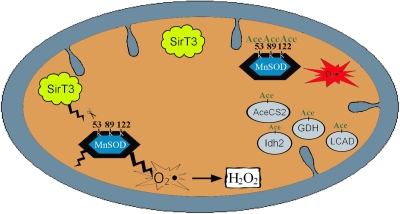
Proposed model figure describing Sirt3 acetylation and subsequent regulation of MnSOD detoxification enzymatic activity. Sirt3 is localized into the inner mitochondrial membrane and appears to be activated by agents that induce oxidative stress, such as ionizing radiation, or changes in cellular nutrient status, such as caloric restriction or fasting. Our data suggests that MnSOD enzymatic activity is directed by acetylation of lysine 122 following fasting or exposure to radiation via the activation of Sirt3. Sirt3 has also been shown to regulate the activity of other mitochondrial proteins including Acetyl-CoA synthetase 2 (AceCS2) [15,44], glutamate dehydrogenase (GDH) [44,45], long-chain acyl-CoA dehydrogenase (LCAD) [46], and isocitrate dehydrogenase 2 (Idh2) [49].

## CONCLUSIONS

The results discussed above suggest that loss of a single mitochondrial protein and the aberrant regulation of the mitochondrial acetylome signaling network that responds to metabolic demands and deacetylates downstream target proteins results in a phenotype permissive for human illnesses associated with aging. In this regard, MnSOD as well as several other recently identified mitochondrial proteins such as acetyl-CoA synthetase [15,44], glutamate dehydrogenase GDH) [44,45], long-chain acyl-CoA dehydrogenase (LCAD) [46], succinate Dehydrogenase [47], Ku70 [20], mitochondrial ribosome subunit MRPL10 [48], and isocitrate dehydrogenase [49]. These Sirt3 targets all appear to regulate critical mitochondrial enzymes that regulate energy metabolism and very strongly suggested that Sirt3 is a mitochondrial metabolism sensing protein maintains energy homeostasis responding to specific nutrient and environmental conditions. As such, we believe the *Sirt3* knockout mice represent a new paradigm that mechanistically links mitochondrial metabolism, the acetylome post-translation signaling network, and age-related disease including ER/PR breast cancer carcinogenesis, radiation-induced liver steatosis, fatty liver, insulin resistance, cardiac hypertrophy, and neurodegenerative diseases.
